# QsvR and OpaR coordinately repress biofilm formation by *Vibrio parahaemolyticus*

**DOI:** 10.3389/fmicb.2023.1079653

**Published:** 2023-02-09

**Authors:** Miaomiao Zhang, Xingfan Xue, Xue Li, Qimin Wu, Tingting Zhang, Wenhui Yang, Lingfei Hu, Dongsheng Zhou, Renfei Lu, Yiquan Zhang

**Affiliations:** ^1^Department of Clinical Laboratory, Affiliated Nantong Hospital 3 of Nantong University, Nantong, Jiangsu, China; ^2^School of Medicine, Jiangsu University, Zhenjiang, Jiangsu, China; ^3^State Key Laboratory of Pathogen and Biosecurity, Beijing Institute of Microbiology and Epidemiology, Beijing, China

**Keywords:** *Vibrio parahaemolyticus*, biofilm, QsvR, OpaR, regulation

## Abstract

Mature biofilm formation by *Vibrio parahaemolyticus* requires exopolysaccharide (EPS), type IV pili, and capsular polysaccharide (CPS). Production of each is strictly regulated by various control pathways including quorum sensing (QS) and bis-(3′–5′)-cyclic di-GMP (c-di-GMP). QsvR, an AraC-type regulator, integrates into the QS regulatory cascade *via* direct control of the transcription of the master QS regulators, AphA and OpaR. Deletion of *qsvR* in wild-type or *opaR* mutant backgrounds altered the biofilm formation by *V. parahaemolyticus*, suggesting that QsvR may coordinate with OpaR to control biofilm formation. Herein, we demonstrated both QsvR and OpaR repressed biofilm-associated phenotypes, c-di-GMP metabolism, and the formation of *V. parahaemolyticus* translucent (TR) colonies. QsvR restored the biofilm-associated phenotypic changes caused by *opaR* mutation, and vice versa. In addition, QsvR and OpaR worked coordinately to regulate the transcription of EPS-associated genes, type IV pili genes, CPS genes and c-di-GMP metabolism-related genes. These results demonstrated how QsvR works with the QS system to regulate biofilm formation by precisely controlling the transcription of multiple biofilm formation-associated genes in *V. parahaemolyticus*.

## Introduction

*Vibrio parahaemolyticus*, a Gram-negative halophilic bacterium, is the primarily causative agent of the seafood-associated gastroenteritis (Broberg et al., 2011). *Vibrio parahaemolyticus* has multiple virulence factors including thermostable direct hemolysin (TDH), type III secretion systems (T3SS1 and T3SS2), and type VI secretion systems (T6SS1 and T6SS2) as well as the capacity to form biofilms (Broberg et al., 2011; Ashrafudoulla et al., 2021). Biofilms are bacterial communities enclosed in an extracellular matrix that endows bacterial cells with a high degree of resistance to stress conditions (Yildiz and Visick, 2009; Ruhal and Kataria, 2021). Biofilm formation requires specific structures including exopolysaccharide (EPS), type IV pili, and capsular polysaccharide (CPS), which are strictly regulated by regulatory cascades such as quorum sensing (QS) and bis-(3′–5′)-cyclic di-GMP (c-di-GMP) signaling (Yildiz and Visick, 2009; Ruhal and Kataria, 2021).

The biofilm matrix contains some chemical components including proteins, EPS, extracellular DNA and membrane vesicles, among which the EPS is the most importnt one (Ruhal and Kataria, 2021). In *V. parahaemolyticus*, the *cpsA-K* and *scvA-O* loci are responsible for EPS biosynthesis (Makino et al., 2003; Liu et al., 2022). These two gene loci are essential for biofilm formation by *V. parahaemolyticus* but may play distinct roles in biofilm-associated colony morphology (Chen et al., 2010; Liu et al., 2022). CPS is associated with opaque (OP)-translucent (TR) colony switching of *V. parahaemolyticus* (Chen et al., 2010). Strains without or with low CPS production manifest the TR phonotype (Chen et al., 2010). CPS has a negative effect on biofilm formation, but both OP and TR strains can form biofilms (Joseph and Wright, 2004; Enos-Berlage et al., 2005). In addition, *V. parahaemolyticus* expresses two kinds of type IV pili, termed mannose-sensitive hemagglutinin (MSHA; encoded by VP2698-2692) and chitin-regulated pilus (ChiRP; encoded by *pilABCD*) (Makino et al., 2003). Both MSHA and ChiRP are important for biofilm formation, but defective biofilms produced by the MSHA mutants can be overcame by extended incubation time (Enos-Berlage et al., 2005; Shime-Hattori et al., 2006). Other structures such as flagella also play roles in biofilm formation by *V. parahaemolyticus* (Enos-Berlage et al., 2005; Li et al., 2020).

The c-di-GMP signaling is widely used by bacteria to modulate biofilm formation, motility, and virulence (Homma and Kojima, 2022). c-di-GMP is synthesized by diguanylate cyclase (DGC) carrying a GGDEF domain and is degraded by phosphodiesterase (PDE) containing EAL or HD-GYP domains (Homma and Kojima, 2022). *V. parahaemolyticus* expresses dozens of GGDEF-or/and EAL-containing proteins (Makino et al., 2003), but only a few of these were investigated. ScrC, which is encoded by *scrABC*, contains both EAL and GGDEF domains (Ferreira et al., 2008; Trimble and McCarter, 2011), but functions as a PDE in the presence of ScrA and ScrB (Ferreira et al., 2008; Trimble and McCarter, 2011). Deletion of *scrABC* enhances biofilm formation but decreases swarming motility (Ferreira et al., 2008; Trimble and McCarter, 2011). ScrG is another EAL- and GGDEF-containing protein that acts only as a PDE in *V. parahaemolyticus* (Kim and McCarter, 2007). Overexpression of *scrG* induces swarming motility but inhibits biofilm formation (Kim and McCarter, 2007). More recently, four GGDEF-type proteins, ScrO, ScrJ, ScrL, and GefA, as well as two EAL-type proteins, LafV and TpdA, were found to be involved in modulation of motility and biofilm formation by *V. parahaemolyticus* (Kimbrough et al., 2020; Kimbrough and McCarter, 2020; Martinez-Mendez et al., 2021; Zhong et al., 2022).

Quorum sensing is a cell density-dependent communication process widely used by bacteria to precisely control gene expression and bacterial behavior in response to changes in concentration of small molecules termed auto-inducers within surroundings (Lu et al., 2018). The QS system of Vibrios generally employs master regulators, AphA and LuxR orthologs, such as OpaR in *V. parahaemolyticus* (Zhang et al., 2012), LuxR in *V. harvei* (Chaparian et al., 2020), HapR in *V. cholerae* (Ball et al., 2017) and SmcR in *V. vulnificus* (Lee et al., 2008), to control gene expression. In general, AphA works at low cell density (LCD) to promote multiple bacterial behaviors including biofilm formation, motility, c-di-GMP synthesis and virulence factor production, whereas the LuxR orthologs function at high cell density (HCD) to inhibit these bacterial behaviors (Rutherford et al., 2011; Wang et al., 2013a,b; Lu et al., 2018, 2019, 2021a,b; Zhang et al., 2017b, 2019, 2021; Sun et al., 2022). In addition, the LuxR orthologs are high expressed at HCD, but they also can be detected at LCD, suggesting that LuxR orthologs function throughout growth (Rutherford et al., 2011; van Kessel et al., 2013; Lu et al., 2019).

QsvR, an AraC-type transcriptional regulator, coordinates with the QS system to control the expression of virulence genes in *V. parahaemolyticus* (Zhang et al., 2019). QsvR directly represses and activates the transcription of *aphA* and *opaR*, respectively (Zhang et al., 2019). AphA indirectly represses *qsvR* transcription at LCD, whereas OpaR indirectly activates its transcription at HCD (Zhang et al., 2019). AphA operates at LCD to activate T3SS1 genes, whereas OpaR and QsvR function at HCD to activate *tdh2* and the T3SS2 genes (Zhang et al., 2019). In addition, QsvR maintains the basal expression of T3SS1 at HCD (Zhang et al., 2019). Moreover, QsvR represses the transcription of *toxR* and *calR*, but activates the transcription of T6SS2 genes, *cpsQ-mfpABC* and *mfpABC* (Qiu et al., 2020; Zhang et al., 2021a,b). Most importantly, the CPS-associated OP-TR transition was regulated by OpaR, which is expressed in OP but not TR strains, with the expression of *opaR* in TR strain converting the TR strain to an OP phenotype (McCarter, 1998). Mutation of *qsvR* in TR strains enhanced initial attachment but impaired biofilm maturation, whereas deletion of *qsvR* in OP strains produced robust biofilms (Enos-Berlage et al., 2005). Therefore, QsvR may coordinate with OpaR to regulate biofilm formation by *V. parahaemolyticus*.

In this study, we demonstrated QsvR works with OpaR to repress biofilm formation and c-di-GMP metabolism, as well as to promote *V. parahaemolyticus* to form OP colonies. OpaR restored biofilm-associated phenotypic changes caused by *qsvR* mutation, and vice versa. Further, QsvR and OpaR worked coordinately to activate the transcription of type IV pili genes, CPS genes, and *scrG*, but repress the transcription of *scrA*. OpaR but not QsvR negatively regulated the transcription of EPS-associated genes. Collectively, our data highlight how QsvR works with the QS system to regulate biofilm formation by precisely controlling the transcription of multiple biofilm formation-associated genes in *V. parahaemolyticus*.

## Materials and methods

### Bacterial strains

*Vibrio parahaemolyticus* strain RIMD2210633 was used as the wild-type (WT) strain in this study (Makino et al., 2003). Non-polar *qsvR* and *opaR* single-gene deletion mutants (*ΔqsvR* and *ΔopaR*), derived from the WT strain, were constructed by our previous studies (Zhang et al., 2012, 2019). The *qsvR* and *opaR* double-gene mutant (*ΔqsvRΔopaR*) was generated *via* deletion of a 615-bp fragment (nucleotides 1–615) of *opaR* from *ΔqsvR* by homologous recombination using suicide plasmid pDS132 (Sun et al., 2012; Zhang et al., 2012).

Complementary plasmids, pBAD33-*qsvR* and pBAD33-*opaR* (Zhang et al., 2012, 2019), were, respectively, introduced into *ΔqsvR* and *ΔopaR*, yielding *ΔqsvR*/pBAD33-*qsvR* (C-*ΔqsvR*), *ΔopaR*/pBAD33-*qsvR*, *ΔqsvR*/pBAD33-*opaR*, and *ΔopaR*/pBAD33-*opaR* (C-*ΔqsvR*). The non-recombinant pBAD33 was transferred into WT and each of the mutants to yield WT/pBAD33, *ΔqsvR*/pBAD33, and *ΔopaR*/pBAD33.

All strains and plasmids used in this study are listed in Supplementary Table S1.

### Bacterial growth conditions

*Vibrio parahaemolyticus* strains were grown similarly as previously described (Zhang et al., 2012; Lu et al., 2019). Briefly, the overnight cell cultures in 2.5% (*w*/*v*) Bacto Heart Infusion (HI) broth (BD Biosciences, United States) were diluted 40-fold into sterile phosphate buffered saline (PBS; pH 7.2), and 150 μl of the diluted cells was spread onto a HI plate with a diameter of 5 cm. Bacterial cells were harvested after 6 h of incubation at 37°C. When necessary, the media were supplemented with 50 μg/ml gentamicin, 5 μg/ml chloramphenicol, or 0.1% (*w*/*v*) arabinose.

### Colony morphology

Colony morphology was performed as previously described (Wang et al., 2013a). Briefly, overnight cell cultures were diluted 50-fold into 5 ml of Difco marine (M) broth 2,216 (BD Biosciences, United States), followed by incubated statically at 30°C for 48 h, and then mixed thoroughly. Two microliter of each culture was spotted on the HI plate and incubated at 37°C for the colony morphology assay.

### Crystal violet staining

Crystal violet staining was performed similarly as previously described (Wang et al., 2013a). Briefly, overnight cell cultures in HI broth were diluted 50-fold into 2 ml of M broth in a 24-well cell culture plate, and incubated at 30°C with shaking at 150 rpm for 48 h. Planktonic cells were collected for determination of OD_600_ values. The surface-attached cells were washed three times with deionized water, and then stained with 0.1% CV, followed by another three washes with deionized water. Bound CV in each well was dissolved with 2.5 ml of 20% ethanol, and the OD_570_ values were determined. Relative biofilm formation was calculated with the formula: OD_570_/OD_600_.

### Detection of OR-TP transition

For detection of OR and TP transition, a small amount of each overnight cell culture in HI broth was streaked onto a HI plate, and then statically incubated at 37°C for 48 h.

### Determination of intracellular c-di-GMP levels

Intracellular c-di-GMP levels were measured as previously described (Gao et al., 2020). Briefly, bacterial cells were harvested from HI plates with 2 ml of ice-cold PBS, incubated at 100°C for 5 min, followed by sonicated for 30 min (power 100%, frequency 37 kHz) in an ice-water bath. After centrifugation, the c-di-GMP concentration in the supernatant was determined with a c-di-GMP enzyme-linked immunosorbent assay (ELISA) kit (Mskbio, China). Total protein concentration in the supernatant was determined by the bicinchoninic acid (BCA) assay. Intracellular c-di-GMP levels were expressed as pmol/mg protein.

### RNA isolation and quantitative PCR (qPCR) analysis

Total RNA was extracted from bacterial cells using TRIzol Reagent (Invitrogen, United States). The cDNA was generated from 1 μg of each RNA sample using a FastKing First Strand cDNA Synthesis Kit (Tiangen Biotech, China) according to the manufacturer’s instructions. The qPCR assay was performed using a LightCycler 480 (Roche, Switzerland) together with SYBR Green master mix (Gao et al., 2011). Expression levels of target genes relative to that of 16S rRNA were determined using the classic 2^−ΔΔCt^ method. All primers used in this study are listed in Supplementary Table S2.

### LacZ fusion and β-galactosidase assay

The regulatory DNA region of each target gene was cloned into the pHRP309 plasmid containing a promoter-less *lacZ* reporter gene and a gentamicin resistance gene (Parales and Harwood, 1993). The recombinant plasmid was transferred into different *V. parahaemolyticus* strains, respectively. The resulting transformants were cultured and then lysed to measure the β-galactosidase activities of the cellular extracts using a β-Galactosidase Enzyme Assay System (Promega, United States) according to the manufacturer’s instructions. The Miller Units representing galactosidase activity were calculated using the following formula: 10^6^ × [(OD_420_ – 1.75 × OD_550_)/(T × V × OD_600_)] (Zhang et al., 2021b). T represents the reaction time (min). T and V represent the reaction time (min) and volume (μL), respectively.

For the two-plasmid reporter assay (Qiu et al., 2020), *E. coli* 100 λpir (Epicentre) bearing a complementary plasmid (pBAD33-*qsvR* or pBAD33-*opaR*) or the empty pBAD33 vector and a recombinant *lacZ* plasmid were cultured in Luria-Bertani (LB) broth at 37°C with shaking at 200 rpm for 12 h. The resultant cultures were diluted 100-fold into 5 ml of fresh LB broth containing 0.1% arabinose and 20 μg/ml chloramphenicol, followed by incubated at 37°C with shaking at 200 rpm till an OD_600_ value of approximately 1.2 was obtained. The *E. coli* cells were harvested and then lysed to measure the β-galactosidase activity in the cell extracts.

### Purification of 6 × His-tagged proteins

The entire coding regions of *opaR* and *qsvR* were individually cloned into the pET28a vector (Novagen, United States). Each recombinant pET28a plasmid was transferred into *E. coli* BL21λDE3 for His-tagged protein expression. Expression and purification of His-QsvR and His-OpaR were performed as previously described (Zhang et al., 2012). The dialyzed proteins were concentrated to approximately 0.5 mg/ml. The purity of the proteins was confirmed by 12% sodium dodecyl sulfate-polyacrylamide gel electrophoresis (SDS-PAGE).

### Electrophoretic mobility-shift assay

Electrophoretic mobility-shift assay (EMSA) was performed as previously described (Zhang et al., 2017b). Briefly, the regulatory DNA region of each target gene was amplified by PCR. The DNA binding assay was performed in a 10 μl reaction volume containing binding buffer [0.5 mM EDTA, 1 mM MgCl_2_, 50 mM NaCl, 0.5 mM DTT, 10 mM Tris–HCl (pH 7.5), and 10 mg/ml salmon sperm DNA], 100 ng target DNA, and increasing amounts of His-tagged protein. After incubation at room temperature for 20 min, the binding products were analyzed in a native 6% (*w*/*v*) polyacrylamide gel with a UV transilluminator after being stained with ethidium bromide (EB) dye.

### Experimental replicates and statistical methods

The qPCR and LacZ fusion were performed at least three independent times with results expressed as means ± standard deviation (SD). A two-way ANOVA with Tukey’s *post hoc* corrections for multiple comparisons was used to calculate statistical significance, with significance accepted at *p* < 0.01. Phenotype assays and EMSA were performed at least three times.

## Results

### QsvR works with OpaR to repress biofilm formation by *Vibrio parahaemolyticus*

**Figure 1:**
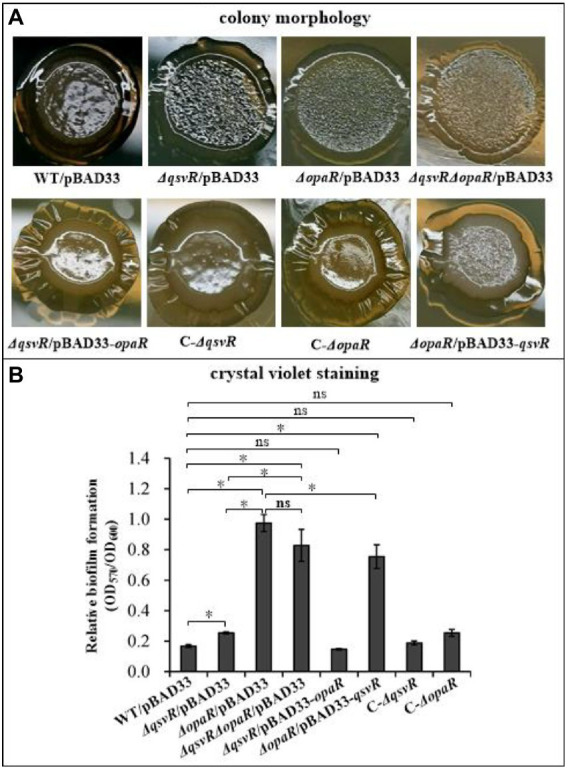
QsvR works with OpaR to repress biofilm formation by *Vibrio parahaemolyticus*. Biofilm formation by *V. parahaemolyticus* strains was assessed by colony morphology **(A)** and intensity of crystal violet staining **(B)**. Photographs are representative of three independent experiments with three replicates each. The asterisks indicate statistical significances (*p* < 0.01). The ‘ns’ means no significant differences (*p* > 0.01).

OpaR repressed *V. parahaemolyticus* biofilm formation by regulation of c-di-GMP metabolism (Zhang et al., 2021a). QsvR also repressed biofilm formation by *V. parahaemolyticus*, but lacks the detailed mechanism (Enos-Berlage et al., 2005). Herein, we constructed single and double-gene mutants of *qsvR* and *opaR* as well as complementary mutants, and which were subjected to colony morphology and CV staining assays (Figure 1). As expected, the colonies of *ΔopaR*/pBAD33 and *ΔqsvR*/pBAD33 are more wrinkled than those of WT/pBAD33, whereas C-*ΔopaR* and C-*ΔqsvR* exhibited a restored phenotypes (Figure 1A). *ΔqsvRΔopaR*/pBAD33 had similar colony morphology to *ΔopaR*/pBAD33 and *ΔqsvR*/pBAD33 (Figure 1A). Most interestingly, *ΔopaR*/pBAD33-*qsvR* and *ΔqsvR*/pBAD33-*opaR* also exhibited restored colony phenotypes (Figure 1A). As further assessed by the CV staining (Figure 1B), *ΔopaR*/pBAD33, *ΔqsvR*/pBAD33, and *ΔqsvRΔopaR*/pBAD33 were more CV positive than WT/pBAD33, whereas C-*ΔopaR*, C-*ΔqsvR*, and *ΔqsvR*/pBAD33-*opaR* exhibited restored CV staining phenotypes. However, *ΔopaR*/pBAD33-*qsvR* had only a partially restored CV staining phenotype compared to *ΔopaR*/pBAD33. *ΔqsvR*/pBAD33 had much less CV staining compared to *ΔopaR*/pBAD33 and *ΔqsvRΔopaR*/pBAD33. *ΔopaR*/pBAD33 and *ΔqsvRΔopaR*/pBAD33 had similar CV staining results. Therefore, OpaR to be more capable than QsvR as an inhibitor of biofilm formation by *V. parahaemolyticus*. Collectively, QsvR worked with OpaR to negatively regulate biofilm formation by *V. parahaemolyticus*.

The *opaR* mutant has no effect on the growth of *V. parahaemolyticus* (Zhang et al., 2012). Herein, we showed that WT, *ΔqsvR* and *ΔopaRΔqsvR* had indistinguishable growth rates in both HI broth and M broth (Supplementary Figure S1), indicating that *qsvR* mutant had no effect on the growth of *V. parahaemolyticus*. Thus, changes in biofilm formation by *qsvR* or/and *opaR* mutants were associated with the regulation of QsvR and OpaR.

### OpaR but not QsvR negatively regulates EPS biosynthesis genes

**Figure 2:**
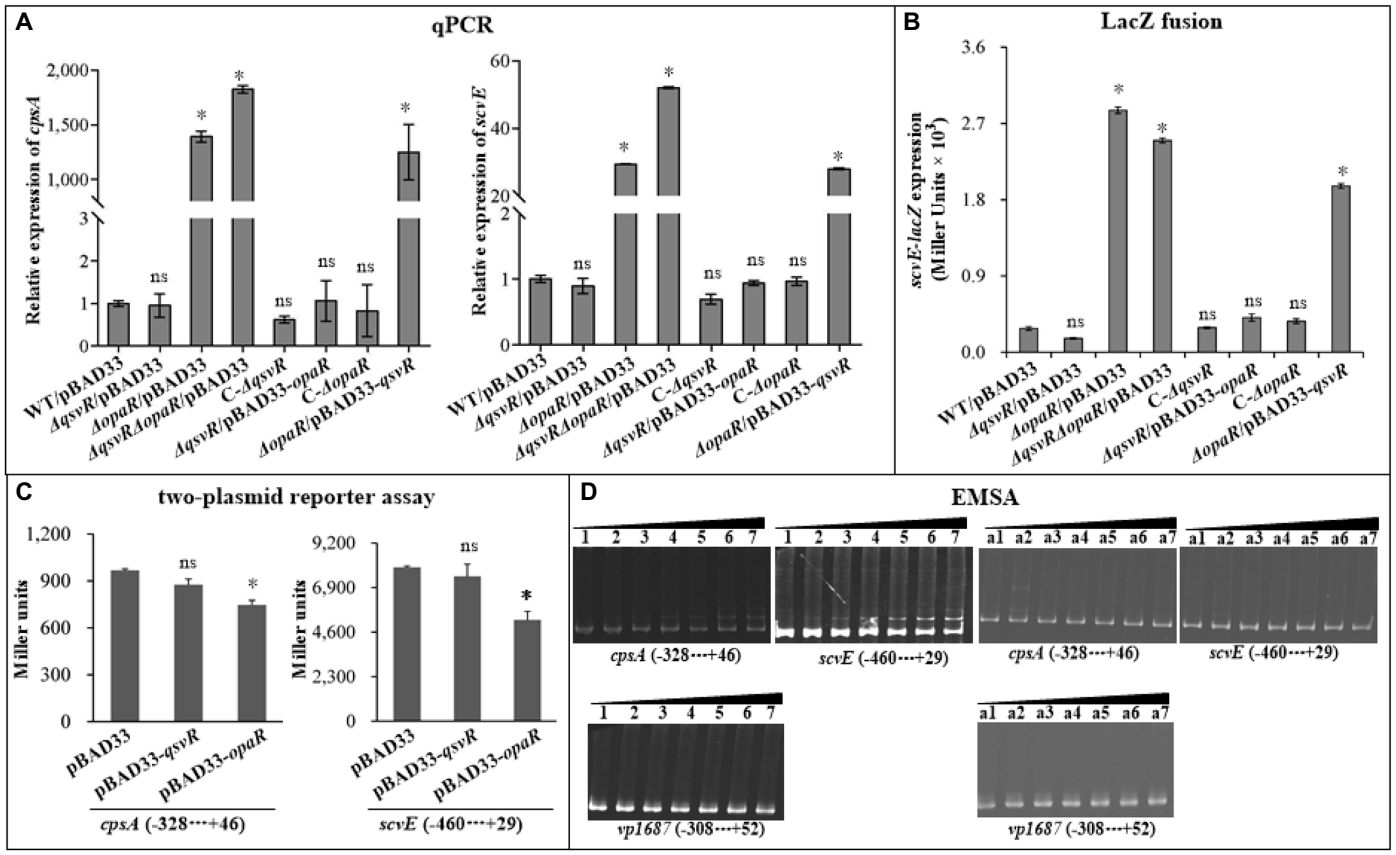
Regulation of *cpsA* and *scvE* by QsvR and OpaR. *V. parahaemolyticus* strains were cultured on HI plates, and bacterial cells were harvested after 6 h of incubation at 37°C. Negative and positive numbers indicate the nucleotide positions upstream and downstream of each target gene, respectively. The asterisks indicate statistical significances relative to WT/pBAD33 or *E. coli* 100 λpir/pBAD33 (*p* < 0.01). The ‘ns’ means no significant differences (*p* > 0.01). **(A)** qPCR. The relative mRNA levels of *cpsA* and *scvE* were examined in the different *V. parahaemolyticus* strains. **(B)** LacZ fusion. The regulatory DNA region of each target gene was cloned into the pHRP309 vector and then transferred into *V. parahaemolyticus* strains to determine the promoter activities (represented by Miller units) in the cellular extracts. **(C)** Two-plasmid reporter assay. The complementary plasmid (pBAD33-*qsvR* or pBAD33-*opaR*) or the empty pBAD33 vector and a recombinant *lacZ* plasmid were simultaneously introduced into *E. coli* 100 λpir (Epicentre), and promoter activities (represented by Miller units) of each target gene in the cellular extracts were determined with a β-Galactosidase Enzyme Assay System (Promega, United States) according to the manufacturer’s instructions. **(D)** Electrophoretic mobility-shift assay (EMSA). The regulatory DNA region of each target gene was incubated with increasing amounts of purified His-QsvR or His-OpaR, and then subjected to 6% (*w*/*v*) polyacrylamide gel electrophoresis. The DNA bands were visualized by EB staining. Lanes 1, 2, 3, 4, 5, 6, and 7 contain 0, 0.4, 0.8, 1.2, 1.4, 2.0, and 2.4 pmol of His-OpaR, respectively. Lanes a1, a2, a3, a4, a5, a6, and a7 contain 0, 0.021, 0.042, 0.063, 0.083, 0.11, and 0.13 pmol of His-QsvR, respectively.

Exopolysaccharide (EPS) production is directly associated with the wrinkled colony phenotype of *V. parahaemolyticus* (Chen et al., 2010). The *cps* and *scv* loci are responsible for EPS synthesis in *V. parahaemolyticus* (Chen et al., 2010; Liu et al., 2022). Herein, qPCR results showed that the mRNA levels of *cpsA* and *scvE* were significantly increased in *ΔopaR*/pBAD33, *ΔqsvRΔopaR*/pBAD33, and *ΔopaR*/pBAD33-*qsvR*. Levels were similar for *ΔqsvR*/pBAD33, C-*ΔqsvR*, C-*ΔopaR*, and *ΔqsvR*/pBAD33-*opaR* compared to WT/pBAD33 (Figure 2A). As further assessed by LacZ fusion assay (Figure 2B), promoter activity of *scrE* was significantly enhanced in *ΔopaR*/pBAD33, *ΔqsvRΔopaR*/pBAD33, and *ΔopaR*/pBAD33-*qsvR*, with no obvious effect in *ΔqsvR*/pBAD33, C-*ΔqsvR*, C-*ΔopaR*, or *ΔqsvR*/pBAD33-*opaR* compared to WT/pBAD33. The LacZ fusion results also demonstrated QsvR to have no regulatory effect on *cpsA* expression, but OpaR positively regulated its expression (unshown data). Previously, OpaR was demonstrated to enhance the promoter activity of *cpsA* (Guvener and McCarter, 2003). Conflicting results between reporter fusion and qPCR assays as well as biofilm phenotypes are difficult to interpret clearly, perhaps due to pHRP309 or its derivative used for the LacZ fusion assay. In addition, the results of two-plasmid reporter assay showed that expression of *opaR* but not *qsvR* from the recombinant pBAD33 significantly lowered promoter activities of *cpsA* and *scvE* (Figure 2C), suggesting that OpaR but not QsvR bound upstream DNA regions of *cpsA* and *scvE* to repress expression in *E. coli*. EMSA results showed that His-OpaR but not His-QsvR bound to upstream DNA fragments of *cpsA* and *scvE* (Figure 2D), but neither bound to the promoter DNA of *vp1687*, which was used as a negative control (Zhang et al., 2019). Taken together, OpaR directly repressed the transcription of *cpsA* and *scvE*, whereas QsvR had no regulatory effect on their expression.

### QsvR works with OpaR to activate the transcription of type IV pili genes

**Figure 3:**
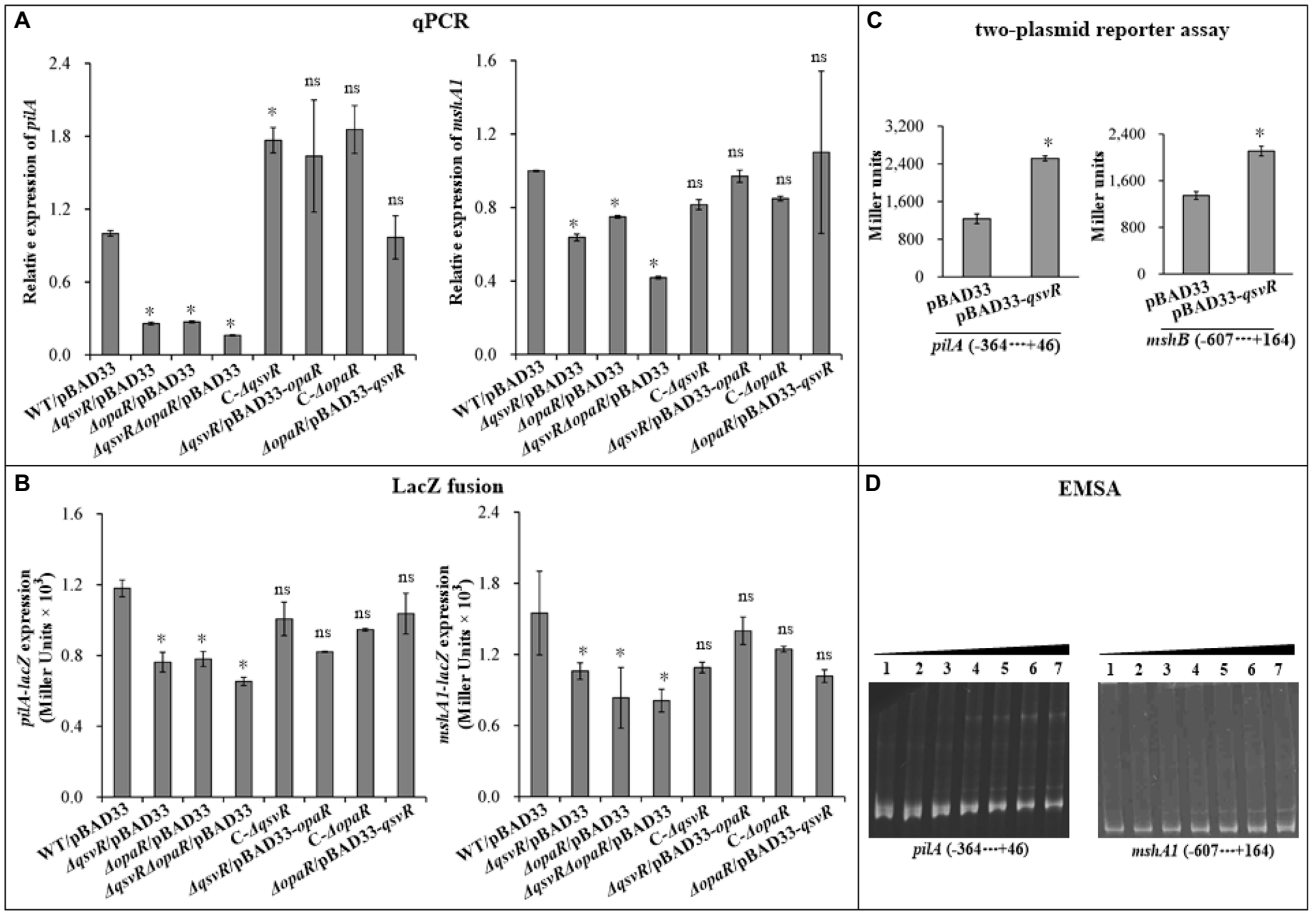
Regulation of *mshA1* and *pilA* by QsvR and OpaR. qPCR **(A)**, LacZ fusion **(B)**, Two-plasmid reporter assay **(C)**, and EMSA **(D)** were carried out as described in Figure 2. Lanes 1, 2, 3, 4, 5, 6, and 7 in the EMSA **(D)** data contain 0, 0.021, 0.042, 0.063, 0.083, 0.11 and 0.13 pmol of His-QsvR, respectively. Negative and positive numbers indicate the nucleotide positions upstream and downstream of each target gene, respectively. The asterisks indicate statistical significances relative to WT/pBAD33 or *E. coli* 100 λpir/pBAD33 (*p* < 0.01). The ‘ns’ means no significant differences (*p* > 0.01).

OpaR directly activated the transcription of MSHA and ChiRP genes (Lu et al., 2021b; Sun et al., 2022). Herein, qPCR results showed that mRNA levels of type IV pili genes, *mshA1* and *pilA*, were significantly reduced in *ΔqsvR*/pBAD33, *ΔopaR*/pBAD33, and *ΔqsvRΔopaR*/pBAD33 relative to WT/pBAD33, with restoration in C-*ΔqsvR*, *ΔqsvR*/pBAD33-*opaR*, C-*ΔopaR*, and *ΔopaR*/pBAD33-*qsvR* (Figure 3A). LacZ fusion assay (Figure 3B) demonstrated reduced *mshA1* and *pilA* promoter activities in *ΔqsvR*/pBAD33, *ΔopaR*/pBAD33, and *ΔqsvRΔopaR*/pBAD33 relative to that in WT/pBAD33, with restoration in C-*ΔqsvR*, *ΔqsvR*/pBAD33-*opaR*, C-*ΔopaR*, and *ΔopaR*/pBAD33-*qsvR*. Previously, OpaR was able to regulate the expression of *mshA1* and *pilA* in *E. coli* (Lu et al., 2021b; Sun et al., 2022). The data presented in Figure 3C demonstrated that expression of *qsvR* in *E. coli* significantly induced the promoter activities of *mshA1* and *pilA*. EMSA demonstrated His-QsvR dose-dependently bind to the upstream DNA fragments of *mshA1* and *pilA* (Figure 3D). Taken together, QsvR worked with OpaR to directly activate the transcription of type IV pili genes in *V. parahaemolyticus*.

### QsvR and OpaR function coordinately to repress c-di-GMP metabolism

**Figure 4:**
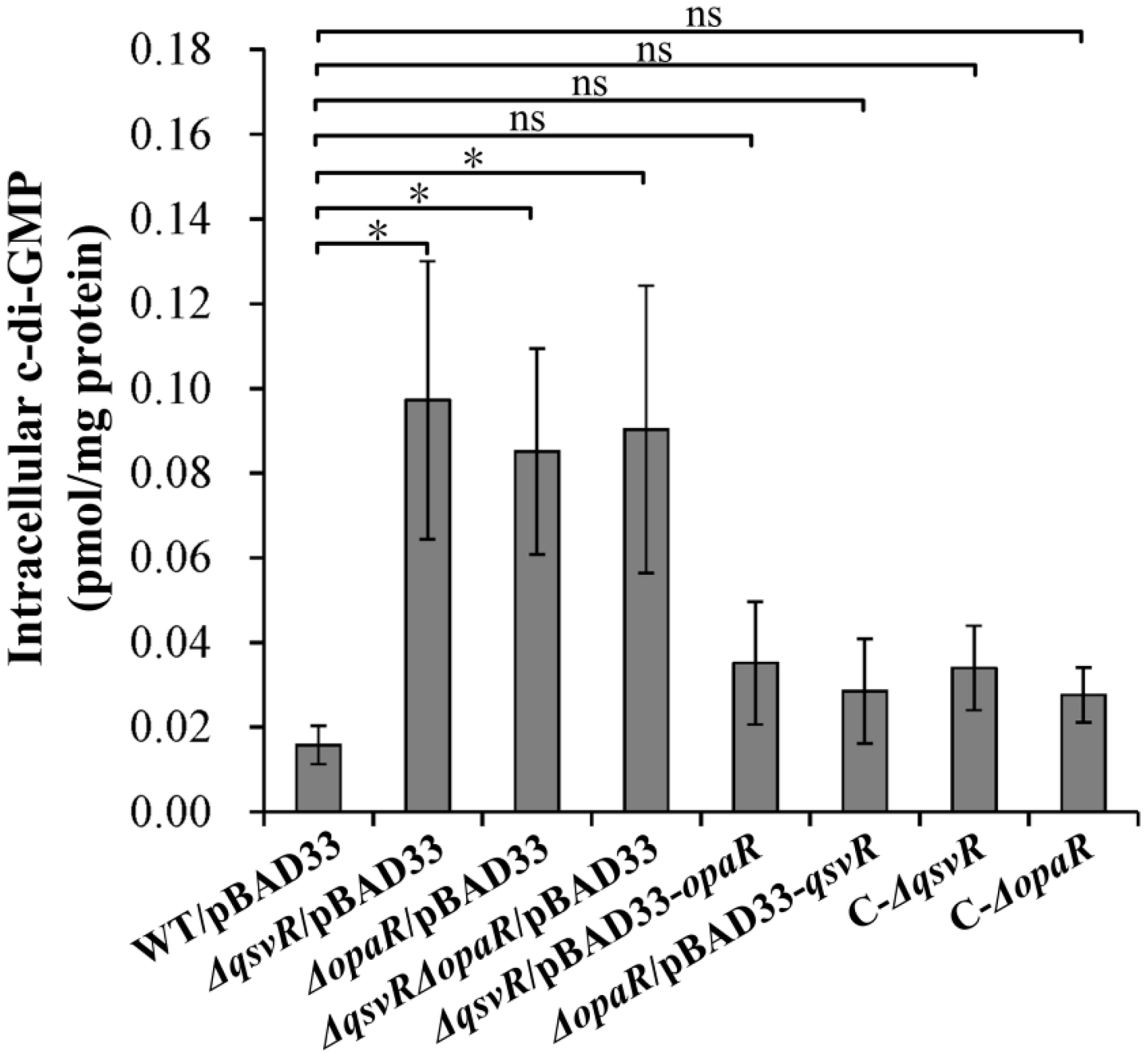
Intracellular c-di-GMP levels in different *V. parahaemolyticus* strains. Bacterial cells were harvested after 6 h of incubation at 37°C on HI plates. c-di-GMP levels were determined using a c-di-GMP enzyme-linked immunosorbent assay (ELISA) kit. The data are expressed as the means ± SD of at least three independent experiments. The asterisks indicate statistical significances relative to WT/pBAD33 (*p* < 0.01). The ‘ns’ means no significant differences (*p* > 0.01).

Elevated intracellular c-di-GMP levels enhance biofilm formation, with deletion of *opaR* increasing the concentration of c-di-GMP in *V. parahaemolyticus* (Zhang et al., 2021a). Herein, intracellular c-di-GMP levels in *ΔqsvR*/pBAD33, *ΔopaR*/pBAD33, and *ΔqsvRΔopaR*/pBAD33 were significantly enhanced relative to WT/pBAD33, with restoration in C-*ΔqsvR*, *ΔqsvR*/pBAD33-*opaR*, C-*ΔopaR*, and *ΔopaR*/pBAD33-*qsvR* (Figure 4). Thus, QsvR and OpaR function coordinately to inhibit c-di-GMP synthesis in *V. parahaemolyticus*.

**Figure 5:**
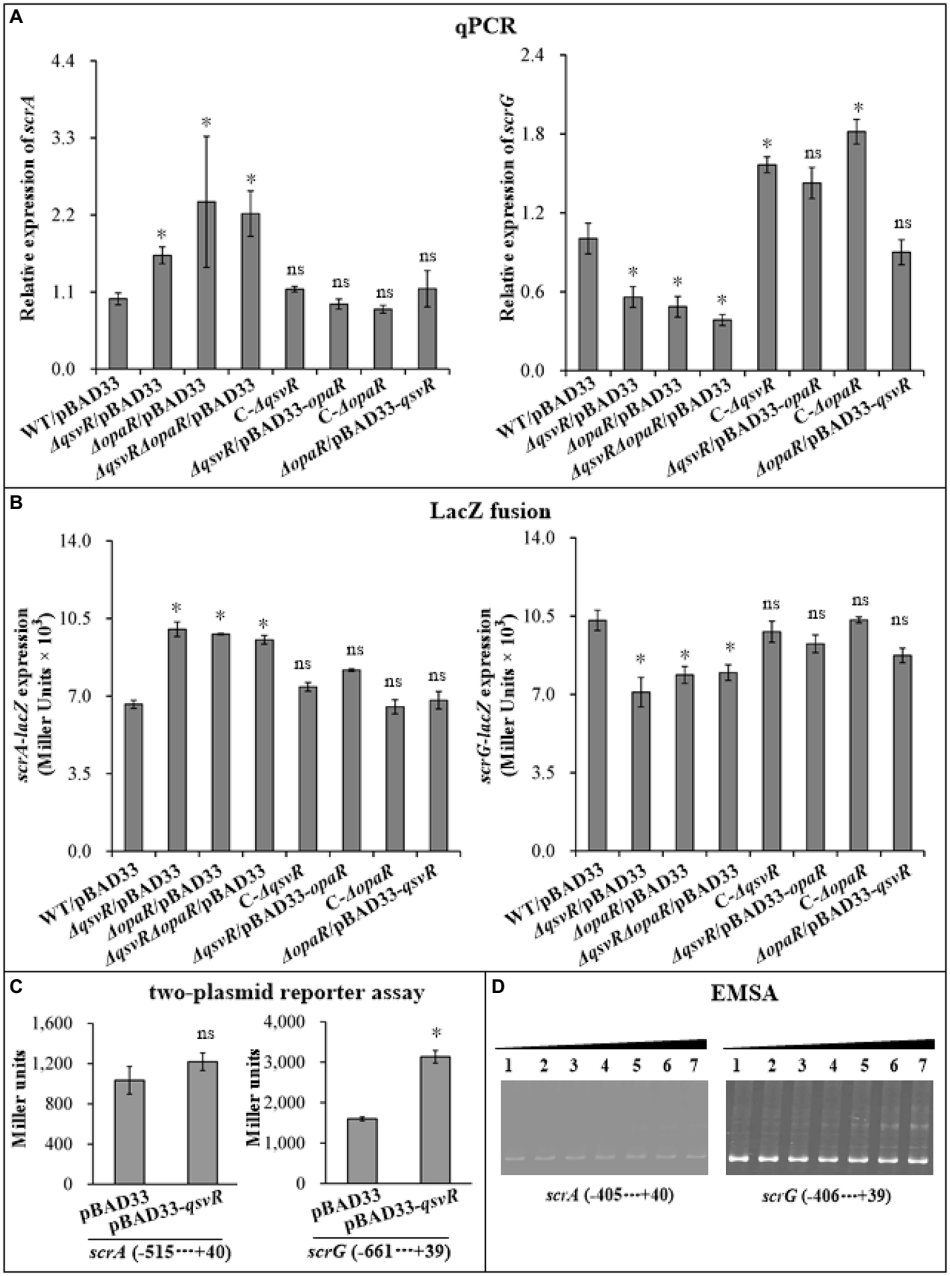
Regulation of *scrG* and *scrA* by QsvR and OpaR. qPCR **(A)**, LacZ fusion **(B)**, Two-plasmid reporter assay **(C)**, and EMSA **(D)** were carried out as described in Figure 2. Lanes 1, 2, 3, 4, 5, 6, and 7 in the EMSA **(D)** data contain 0, 0.021, 0.042, 0.063, 0.083, 0.11, and 0.13 pmol of His-QsvR, respectively. Negative and positive numbers indicate the nucleotide positions upstream and downstream of each target gene, respectively. The asterisks indicate statistical significances relative to WT/pBAD33 or *E. coli* 100 λpir/pBAD33 (*p* < 0.01). The ‘ns’ means no significant differences (*p* > 0.01).

Two well-studied c-di-GMP metabolism-associated genes, *scrA* (the first gene of the *scrABC* operon) and *scrG*, were selected as target genes to investigate QsvR-and OpaR-mediated gene regulation. qPCR results showed that the mRNA levels of *scrA* and *scrG* were significantly increased and decreased, respectively, in *ΔqsvR*/pBAD33, *ΔopaR*/pBAD33, and *ΔqsvRΔopaR*/pBAD33 relative to that in WT/pBAD33, with restoration in C-*ΔqsvR*, *ΔqsvR*/pBAD33-*opaR*, C-*ΔopaR*, and *ΔopaR*/pBAD33-*qsvR* (Figure 5A). Similarly, LacZ fusion assays showed that the promoter activity of *scrA* or *scrG* was significantly enhanced and reduced, respectively, in *ΔqsvR*/pBAD33, *ΔopaR*/pBAD33, and *ΔqsvRΔopaR*/pBAD33 relative to that in WT/pBAD33, with restoration in C-*ΔqsvR*, *ΔqsvR*/pBAD33-*opaR*, C-*ΔopaR*, and *ΔopaR*/pBAD33-*qsvR* (Figure 5B). Furthermore, OpaR repressed and activated the promoter activity of *scrA* and *scrG* in a heterologous host, respectively (Zhang et al., 2021a). Similar results were observed for QsvR regulation of *scrG*, with no apparent regulatory effect on *scrA* expression in *E. coli* (Figure 5C). EMSA demonstrated that His-QsvR dose-dependently bound to the upstream DNA fragment of *scrG* but not *scrA* (Figure 5D). Taken together, QsvR worked with OpaR to repress c-di-GMP synthesis in *V. parahaemolyticus*, likely due to direct control of the transcription of c-di-GMP metabolism-related genes.

### QsvR and OpaR work coordinately to regulate the OP-TR transition of *Vibrio parahaemolyticus*

**Figure 6:**
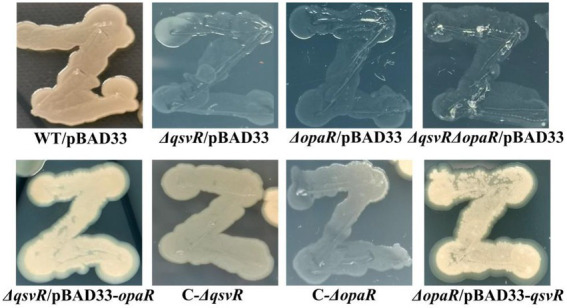
The opaque (OP)-translucent (TR) transition of *V. parahaemolyticus* strains. *V. parahaemolyticus* strains were grown in M broth at 30°C with shaking at 200 rpm overnight, and a small amount of each cell culture was taken with an inoculation loop, streaked directly on a HI plate, and then statically incubated at 37°C for 48 h.

Previously, OpaR was shown to regulate the OP-TR transition (McCarter, 1998), but lacks the detailed mechanisms. Herein, the data showed that the TR cell type was exhibited by *ΔqsvR*/pBAD33, *ΔopaR*/pBAD33, and *ΔqsvRΔopaR*/pBAD33, whereas WT/pBAD33 exhibited the OP cell type (Figure 6). C-*ΔqsvR*, *ΔqsvR*/pBAD33-*opaR*, C-*ΔopaR*, and *ΔopaR*/pBAD33-*qsvR* exhibited the restored OP cell type (Figure 6). Therefore, QsvR and OpaR functioned coordinately to regulate the OP–TR transition of *V. parahaemolyticus*.

**Figure 7:**
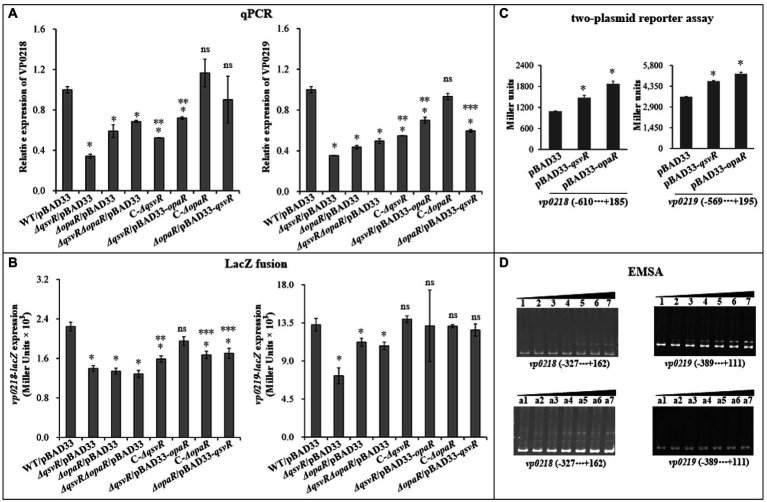
Regulation of *vp0218* and *vp0218* by QsvR and OpaR. qPCR **(A)**, LacZ fusion **(B)**, Two-plasmid reporter assay **(C)**, and EMSA **(D)** were carried out as described in Figure 2. Lanes 1, 2, 3, 4, 5, 6, and 7 in the EMSA **(D)** data contain 0, 0.4, 0.8, 1.2, 1.4, 2.0, and 2.4 pmol of His-OpaR, respectively. Lanes a1, a2, a3, a4, a5, a6, and a7 contain 0, 0.021, 0.042, 0.063, 0.083, 0.11, and 0.13 pmol of His-QsvR, respectively. Negative and positive numbers indicate the nucleotide positions upstream and downstream of each target gene, respectively. The single asterisk symbol (*) indicates statistical significances relative to WT/pBAD33 or *E. coli* 100 λpir/pBAD33 (*p* < 0.01). The double asterisk symbol (**) indicates statistical significances relative to *ΔqsvR*/pBAD33 (*p* < 0.01). The three asterisk symbol (***) indicates statistical significances relative to *ΔopaR*/pBAD33 (*p* < 0.01). The ‘ns’ means no significant differences relative to WT/pBAD33 (*p* > 0.01).

OP and TR cell types directly relate to CPS production (Chen et al., 2010). The *vp0215*-*0237* gene cluster is responsible for CPS synthesis in *V. parahaemolyticus* (Chen et al., 2010). The *vp0215*-*0237* locus contains at least two operons, *vp0218*-*0215* and *vp0219*-*0237* (Chen et al., 2010). *vp0218* and *vp0219* are transcribed in opposite directions and share an intergenic region 601 bp in length (Makino et al., 2003; Chen et al., 2010). In this study, *vp0218* and *vp0219* were selected as target genes to assess QsvR-and OpaR-mediated gene regulation. qPCR showed that mRNA levels of *vp0218* and *vp0219* were significantly decreased in *ΔqsvR*/pBAD33, *ΔopaR*/pBAD33, and *ΔqsvRΔopaR*/pBAD33 relative to WT/pBAD33, with restoration in C-*ΔqsvR*, *ΔqsvR*/pBAD33-*opaR*, C-*ΔopaR*, and *ΔopaR*/pBAD33-*qsvR* (Figure 7A). LacZ fusion assay showed that the promoter activities of *vp0218* and *vp0219* were significantly reduced in *ΔqsvR*/pBAD33, *ΔopaR*/pBAD33, and *ΔqsvRΔopaR*/pBAD33 relative to WT/pBAD33, with restoration in C-*ΔqsvR*, *ΔqsvR*/pBAD33-*opaR*, C-*ΔopaR*, and *ΔopaR*/pBAD33-*qsvR* (Figure 7B). In addition, both QsvR and OpaR were able to induce the expression of *vp0218* and *vp0219* in a heterologous host (Figure 7C). By EMSA (Figure 7D), both His-QsvR and His-OpaR were able to dose-dependently bind to the regulatory DNA fragments of *vp0218* and *vp0219*. Therefore, both QsvR and OpaR were able to directly activate the transcription of *vp0218* and *vp0219*.

Taken together, these results demonstrated QsvR and QsvR to function coordinately to regulate the OP–TR transition of *V. parahaemolyticus* by directly activating the transcription of CPS synthesis genes.

### No interplay of QsvR and OpaR at target promoters

**Figure 8:**
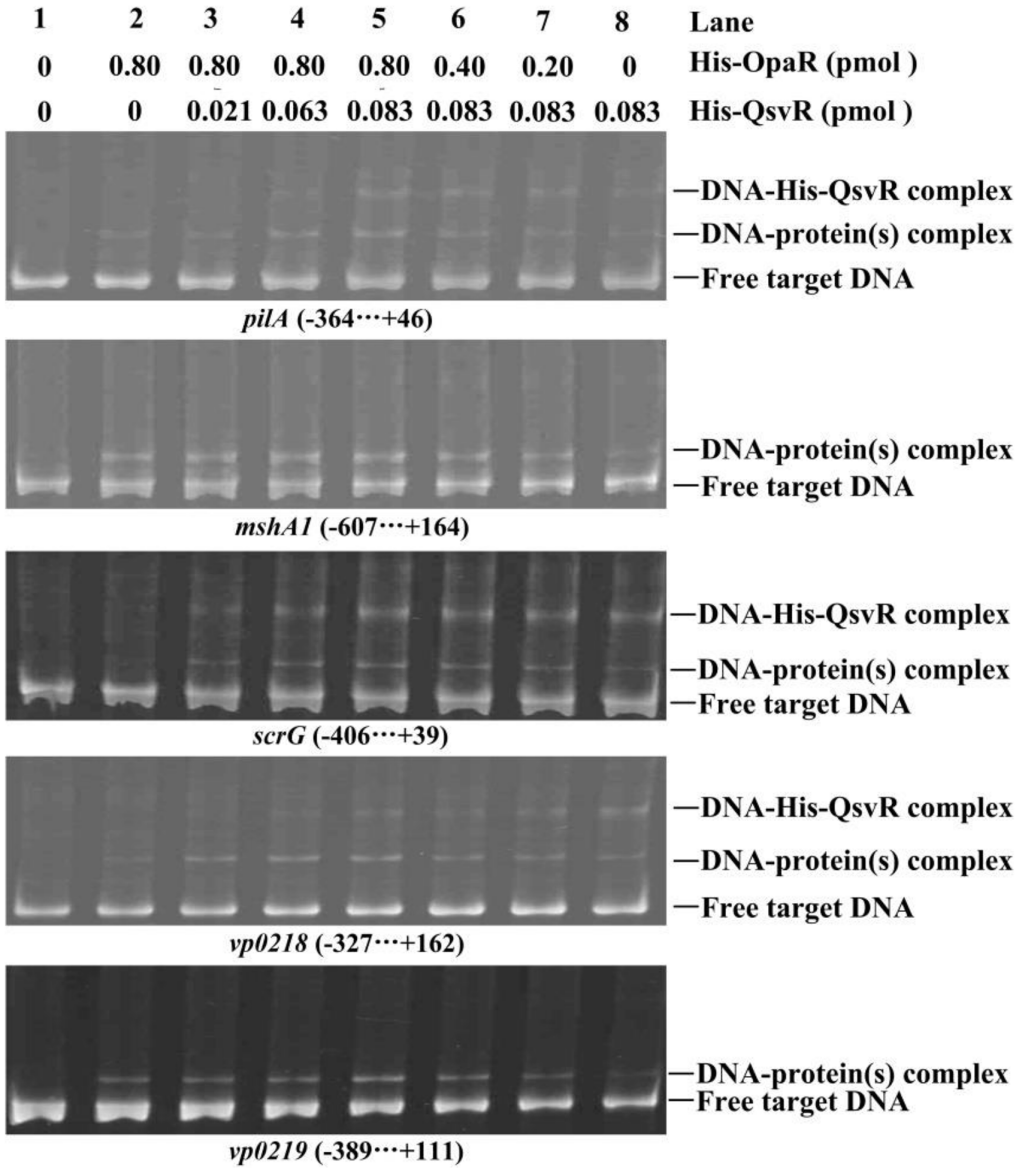
Competitive DNA-binding assays. The experiments were carried out as described in Figure 2D.

As confirmed in this work and as previously reported, both QsvR and OpaR directly regulate the transcription of *pilA*, *mshA1*, *scrG*, *vp0218*, and *vp0219* (Lu et al., 2021b; Zhang et al., 2021a; Sun et al., 2022). To determine whether QsvR promoter binding affects OpaR and vice versa, we performed competitive EMSA using the promoter-proximal DNA fragments of *pilA*, *mshA1*, *scrG*, *vp0218*, and *vp0219* with varying amounts of His-QsvR and His-OpaR (Figure 8). The retarded bands of DNA-His-QsvR and DNA-His-OpaR complexes overlapped each other, although the retarded bands of individual proteins (lane 2 or 8) were much weaker than those of the mixed proteins (lanes 3–7). The most apparent retarded bands were found in the lane with the greatest amount of both proteins (lane 5). A second dose-dependent retarded band of DNA-His-QsvR was also observed for some target genes, e.g., *vp0218*. These results suggested no competitive binding by QsvR and OpaR for these regulatory DNA fragments.

## Discussion

OpaR controls approximately 15% of *V. parahaemolyticus* genes, including biofilm-related genes (Gode-Potratz and McCarter, 2011; Kernell Burke et al., 2015). OpaR regulates CPS-associated OP-TR transition of *V. parahaemolyticus*, and expression of OpaR in TR strains converts those strains to an OP phenotype (McCarter, 1998). Deletion of *qsvR* in either OP (WT) or TR (*opaR* mutant) strains alters their ability to form biofilms even though the mechanisms may differ (Enos-Berlage et al., 2005). Herein, we showed that deletion of *qsvR* in both WT and *ΔopaR* backgrounds produced similar colony phenotypes to *ΔopaR*, all of which were more wrinkled than that of WT (Figure 1A). Both QsvR and OpaR acted as negative regulators of biofilm formation by *V. parahaemolyticus* RIMD2210633, but their regulatory activities were not additive, with OpaR a stronger inhibitor than QsvR. For *V. parahaemolyticus* BB22OP *ΔqsvR* and *ΔqsvRΔopaR* have a similar capacity to form biofilms, with colonies that are not rough and resembled their parental colony type (Enos-Berlage et al., 2005). We have observed similar contradictory results for these two strains, which was attributed to large differences in the two genomes (Jensen et al., 2013; Zhang et al., 2021a). Most interestingly, expression of *opaR* in *ΔqsvR* restored the phenotype of *ΔqsvR* and vice versa, suggesting that OpaR and QsvR may substitute for each other in biofilm formation.

The *cps* and *scv* loci are responsible for EPS production, which is directly associated with wrinkled colonies and enhanced biofilm formation by *V. parahaemolyticus* (Chen et al., 2010; Liu et al., 2022). Herein, OpaR directly repressed the transcription of *cpsA* and *scvE*, whereas QsvR had no effect on their expression (Figure 2). A previous study showed that QsvR did not control *cpsA* transcription in either OP or TR backgrounds (Enos-Berlage et al., 2005). These results may explain why OpaR had a greater capacity for the inhibition of biofilm formation by *V. parahaemolyticus* RIMD2210633 than did QsvR. Previously, CpsS was shown to repress *cpsR* transcription, followed by CpsR activation of *cpsQ* transcription, with CpsQ repression of *cpsS* transcription but activation of *cpsA* (Guvener and McCarter, 2003; Ferreira et al., 2012). CpsR alone increases *cps* gene transcription in a *scrAΔopaR* background (Guvener and McCarter, 2003). Both QsvR and OpaR activate *cpsQ* transcription (Zhou et al., 2013; Zhang et al., 2021b). In addition, both H-NS and ToxR activate EPS production (Chen et al., 2018; Zhang et al., 2018). AphA is also required for the expression of the *scv* locus (Chen et al., 2022; Liu et al., 2022). Furthermore, overexpression of *scrABC* or *scrG* inhibits *cpsA* transcription (Boles and McCarter, 2002; Kim and McCarter, 2007). Therefore, expression of EPS-related genes is tightly controlled by regulatory networks composed of various transcriptional regulators and signaling pathways. The present data provided for a better understanding of the regulatory networks of EPS-associated genes.

Type IV pili play critical roles in biofilm formation and bacterial colonization (Yildiz and Visick, 2009; O'Boyle et al., 2013). Expression of MSHA and ChiRP is induced by OpaR in *V. parahaemolyticus* RIMD2210633 (Lu et al., 2021b; Sun et al., 2022). Herein, we demonstrated QsvR to activate transcription of *mshA1* and *pilA* in the WT background but not in a the *ΔopaR* background (Figure 3). However, expression of *qsvR* in *ΔopaR* restored expression of *mshA1* and *pilA*, and vice versa (Figure 3). Both QsvR and OpaR bound to the upstream DNA fragments of *mshA1* and *pilA* (Figure 3; Lu et al., 2021b; Sun et al., 2022). Binding sequences of QsvR are typically long and AT-rich (Zhang et al., 2019, 2021b; Qiu et al., 2020). However, there was no competitive binding between QsvR and OpaR for the regulatory DNA regions of *mshA1* and *pilA* (Figure 8). Roles for type IV pili in *V. parahaemolyticus* RIMD2210633 have been well studied (Enos-Berlage et al., 2005; Shime-Hattori et al., 2006; Yildiz and Visick, 2009; O'Boyle et al., 2013), but a detailed understanding of their expression regulation requires more investigation in the future.

The c-di-GMP content in bacteria influences biofilm formation and motility (Yildiz and Visick, 2009). Deletion of *qsvR* in the WT background enhanced intracellular c-di-GMP levels, whereas *qsvR* deletion in the *ΔopaR* background did not influence intracellular c-di-GMP levels (Figure 4). OpaR negatively regulates c-di-GMP production in *V. parahaemolyticus* RIMD2210633 (Zhang et al., 2021a). Expression of *qsvR* in *ΔopaR* led to restored intracellular c-di-GMP levels, and vice versa (Figure 4). *V. parahaemolyticus* RIMD2210633 harbors dozens of genes encoding proteins that may be required for c-di-GMP metabolism, but only a few (including *scrABC* and *scrG*) are studied (Boles and McCarter, 2002; Makino et al., 2003; Kim and McCarter, 2007; Ferreira et al., 2008, 2012). OpaR directly regulate several putative c-di-GMP metabolism-associated genes including *scrABC* and *scrG* (Zhang et al., 2021a). Herein, we showed that QsvR directly activated *scrG* transcription but indirectly repressed *scrA* transcription (Figure 5). Expression of *qsvR* in *ΔopaR* restored expression levels of *scrG* and *scrA*, and vice versa (Figure 5). It is noteworthy that QsvR inhibits biofilm formation and c-di-GMP production as well as directly regulates the transcription of *scrABC* and *scrG* but exerts no regulatory effect on the expression of EPS genes. Biofilm formation by *V. parahaemolyticus* is uncorrelated with the EPS content in the biofilm matrix (Li et al., 2020), but it is still hard to clearly explain these contradictions. Perhaps there exist additional regulators that complement the effect on EPS production in the *qsvR* mutant. Moreover, QsvR integrates into the QS cascade to regulate gene expression *via* direct regulation of the master QS regulators, AphA and OpaR (Zhang et al., 2019). QS and c-di-GMP signals are two crucial regulatory cascades. Thus, the direct association between QsvR and OpaR regulation and c-di-GMP metabolism may be beneficial for *V. parahaemolyticus* to precisely control bacterial behaviors.

OpaR-dependent CPS production has been reported (McCarter, 1998), but lacks detailed mechanisms. Herein, we demonstrated that the *qsvR* mutants in WT or *ΔopaR* backgrounds exhibited TR cell type morphologies, whereas obviously no difference were observed for *ΔopaR* and *ΔqsvRΔopaR* (Figure 6). Expression of *qsvR* in *ΔopaR* restored an OP cell type, and vice versa (Figure 6). QsvR worked with OpaR to coordinately promote CPS production *via* directly activation of the transcription of CPS-associated genes in *V. parahaemolyticus*. A role for CPS in biofilm formation has not been described for *V. parahaemolyticus*, and future studies should address this issue.

In conclusion, this study demonstrated QsvR and OpaR to work coordinately to repress biofilm-associated phenotypes and c-di-GMP metabolism, and as well to promote *V. parahaemolyticus* OP colony formation. QsvR restored biofilm-associated phenotypic changes caused by the *opaR* mutation, and vice versa. Furthermore, QsvR and OpaR were shown to work coordinately to activate the transcription of type IV pili genes, CPS genes, and *scrG*, as well as to repress *scrA* transcription. In addition, OpaR but not QsvR negatively regulated the transcription of *cps* and *scv* genes. Thus, our data highlight how QsvR works with the QS system to regulate biofilm formation by precisely controlling the transcription of multiple biofilm formation-associated genes in *V. parahaemolyticus*.

## Data availability statement

The raw data supporting the conclusions of this article will be made available by the authors, without undue reservation.

## Author contributions

MZ, XX, XL, QW, TZ, WY, and LH performed the laboratory experiments and analyzed the results. YZ, RL, and DZ designed, organized and supervised the experiments. MZ and YZ drafted the manuscript. All authors contributed to the article and approved the submitted version.

## Funding

This work was supported by the National Natural Science Foundation of China (grant no. 82072239) and the Natural Science Research Project of Nantong Science and Technology Bureau (grant no. JC2021027).

## Conflict of interest

The authors declare that the research was conducted in the absence of any commercial or financial relationships that could be construed as a potential conflict of interest.

## Publisher’s note

All claims expressed in this article are solely those of the authors and do not necessarily represent those of their affiliated organizations, or those of the publisher, the editors and the reviewers. Any product that may be evaluated in this article, or claim that may be made by its manufacturer, is not guaranteed or endorsed by the publisher.

## Supplementary material

The Supplementary material for this article can be found online at: https://www.frontiersin.org/articles/10.3389/fmicb.2023.1079653/full#supplementary-material
