# Supplementary material for: QsvR and OpaR coordinately repress biofilm formation by Vibrio parahaemolyticus
Source: Front Microbiol. 2023 Feb 9;14:1079653. doi: 10.3389/fmicb.2023.1079653 (PMC9948739; doi:10.3389/fmicb.2023.1079653)
Supplement: Supplementary file 1 [file Data_Sheet_1.docx]

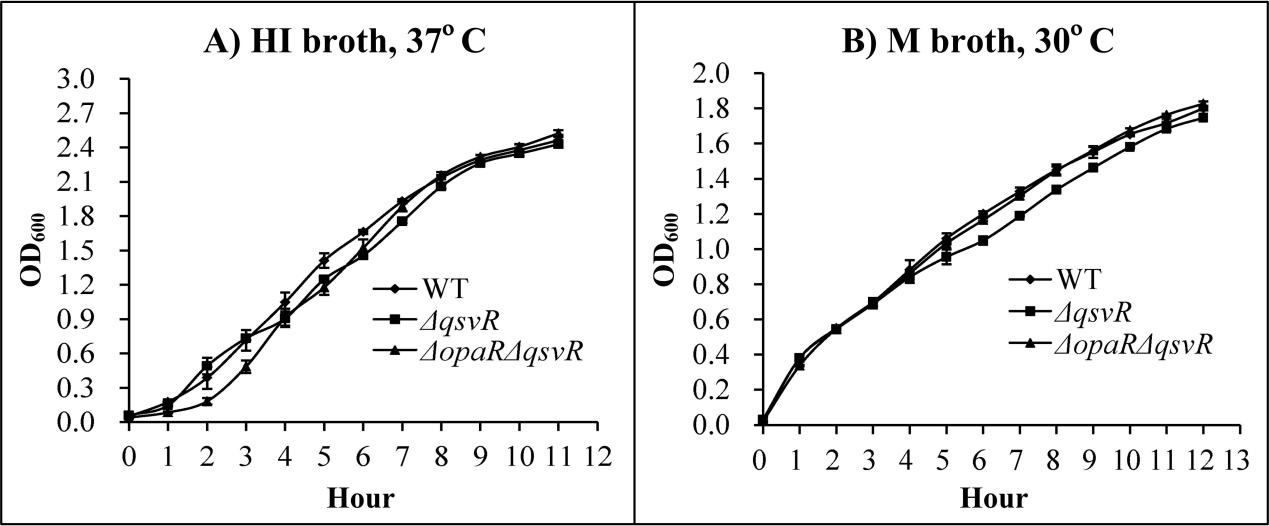


**Supporting information Fig. 1 Growth curves of WT, *ΔqsvR* and *ΔqsvRΔopaR***. WT, *ΔqsvR* and *ΔqsvRΔopaR* were grown in HI broth at 37ºC or in M broth at 30ºC with shaking at 200 rpm. OD_600_ values for each strain were measured at 1 h intervals. Experiments were performed at least twice with three replicates per trial for each strain.
