# Supplementary material for: QsvR and OpaR coordinately repress biofilm formation by Vibrio parahaemolyticus
Source: Front Microbiol. 2023 Feb 9;14:1079653. doi: 10.3389/fmicb.2023.1079653 (PMC9948739; doi:10.3389/fmicb.2023.1079653)
Supplement: Supplementary file 2 [file Data_Sheet_2.docx]

**Table S1. Bacterial strains and plasmids used in this study**

| **Strains** | **Characteristics** | **Source** |
| --- | --- | --- |
| ***V. parahaemolyticus*** RIMD2210633 | | |
| *V. parahaemolyticus* RIMD2210633, Wild-type (WT) | A pandemic O3:K6 strain isolated from a patient with traveller’s diarrhoea in 1996 | [1] |
| *ΔqsvR* | RIMD2210633 *qsvR*^-^ (deletion of the nucleotides from 101 to 710 of *qsvR*) | [2] |
| *ΔopaR* | RIMD2210633 *opaR*^-^ (deletion of the nucleotides from 1 to 615 bp of *opaR*) | [3] |
| *ΔqsvRΔopaR* | RIMD2210633 *qsvR*^-^ *opaR*^-^ (deletion of the nucleotides from 101 to 710 of *qsvR* and from 1 to 615 bp of *opaR*) | This study |
| C-*ΔqsvR* | The *qsvR* mutant with a PCR fragment covering the coding region of *qsvR* in the *Sac* I/*Xba* I sites of pBAD33 | [2] |
| C-*ΔopaR* | The *opaR* mutant with a PCR fragment covering the coding region of *opaR* in the *Xba* I/*Hind* III sites of pBAD33 | [2] |
| *ΔqsvR/*pBAD33-*opaR* | The *qsvR* mutant with a PCR fragment covering the coding region of *opaR* in the *Xba* I/*Hind* III sites of pBAD33 | This study |
| *ΔopaR/*pBAD33-*qsvR* | The *opaR* mutant with a PCR fragment covering the coding region of *qsvR* in the *Sac* I/*Xba* I sites of pBAD33 | This study |
| WT/pBAD33 | The WT strain harboring the nonrecombinant pBAD33 | [2] |
| *ΔqsvR*/pBAD33 | The *qsvR* mutant harboring the nonrecombinant pBAD33 | [2] |
| *ΔopaR*/pBAD33 | The *opaR* mutant harboring the nonrecombinant pBAD33 | [2] |
| *ΔqsvRΔopaR*/pBAD33 | The *qsvR* and *opaR* double gene mutant harboring the nonrecombinant pBAD33 | This study |
| ***E. coli*** | | |
| 100λpir | TransforMax EC100D, electrocompetent *E. coli* | Epicentre |
| BL21 (λDE3) | His-tagged protein expression | Novagen |
| **Plasmids** | | |
| pBAD33 | Cm^r^, a cloning vector containing the p15A origin of replication | [4] |
| pHRP309 | Broad-host-range *lacZ* expression vector | [5] |

**References**

1. Makino K, Oshima K, Kurokawa K, Yokoyama K, Uda T, Tagomori K, Iijima Y, Najima M, Nakano M, Yamashita A *et al*: **Genome sequence of Vibrio parahaemolyticus: a pathogenic mechanism distinct from that of V cholerae**. *Lancet* 2003, **361**(9359):743-749.

2. Zhang Y, Hu L, Qiu Y, Osei-Adjei G, Tang H, Zhang Y, Zhang R, Sheng X, Xu S, Yang W *et al*: **QsvR integrates into quorum sensing circuit to control Vibrio parahaemolyticus virulence**. *Environ Microbiol* 2019, **21**(3):1054-1067.

3. Zhang Y, Qiu Y, Tan Y, Guo Z, Yang R, Zhou D: **Transcriptional regulation of opaR, qrr2-4 and aphA by the master quorum-sensing regulator OpaR in Vibrio parahaemolyticus**. *PLoS One* 2012, **7**(4):e34622.

4. Sun F, Zhang Y, Qiu Y, Yang H, Yang W, Yin Z, Wang J, Yang R, Xia P, Zhou D: **H-NS is a repressor of major virulence gene loci in Vibrio parahaemolyticus**. *Front Microbiol* 2014, **5**:675.

5. Parales RE, Harwood CS: **Construction and use of a new broad-host-range lacZ transcriptional fusion vector, pHRP309, for gram- bacteria**. *Gene* 1993, **133**(1):23-30.

**Table S2. Oligonucleotide primers used in this study**

| **Target** | **Primers (forward/reverse, 5'-3')** | |
| --- | --- | --- |
| **Construction of mutants** | | |
| *opaR* | GTGACTGCAGACTGCCTTGGTAACGCTCTG  /GTTCGTGTTCAAATCTGAGCTATCCATTTTCCTTGCCATTTG | |
|  | CAAATGGCAAGGAAAATGGATAGCTCAGATTTGAACACGAAC  /GTGAGCATGCATGGGCTGCATCAGGTCG | |
|  | GTGACTGCAGACTGCCTTGGTAACGCTCTG  /GTGAGCATGCATGGGCTGCATCAGGTCG | |
| *qsvR* | GTGACTGCAGATGCTAAAAGCGGTGATTC  /GATTCAAATGCGATTTCTGTTGGCTGGTGGACGACTAATG | |
|  | CATTAGTCGTCCACCAGCCAACAGAAATCGCATTTGAATC  /GTGAGCATGCGAGAAGTCTGTAAACGAAACG | |
|  | GTGACTGCAGATGCTAAAAGCGGTGATTC  /GTGAGCATGCGAGAAGTCTGTAAACGAAACG | |
| **Construction of complemented mutants** | | |
| *opaR* | GATTCTAGAAGGAGGAATTCACCATGGACTCAATTGCAAAGAG  /GACAAGCTTTTAGTGTTCGCGATTGTAG | |
| *qsvR* | GATTCTAGAAGGAGGAATTCACCATGCCGAACATTGAGATCATTC  /GACAAGCTTTTAACCTCTTACTACCTGATTACG | |
| **Protein expression** | | |
| *opaR* | AGCGGGATCCATGGACTCAATTGCAAAGAG  /AGCGAAGCTTTTAGTGTTCGCGATTGTAG | |
| *qsvR* | AGCGGGATCCATGCCGAACATTGAGATCATTC  /AGCGAAGCTTTTAACCTCTTACTACCTGATTACG | |
| **qPCR** | | |
| *cpsA* | GAGAGCGGCAACCTATATCG/CGCCACGCCAACAGTAATG | |
| *scvE* | GACAGGTCGTGATGCCATTC/GGCGATGATGACCGAAGTG | |
| *pilA* | TACACCGCCACCCATAACG/AGCCATTCTCGCCAGGTATG | |
| *pilA* | GGCTGAGCTGCATTACCAAG/TCCCACCGTCGATAGAACTG | |
| *mshA1* | GCGATTGATGGTGCTTCTG/GCCCAATCTTCATCCAAACC | |
| *scrA* | CACACCACGAACACATTGC/TCAATAGCGTCACGGAATGC | |
| *scrG* | AAGCCGTGGTGGAAGAAGG/GCGTGTTGAGTGCGTTGG | |
| *vp0218* | CTCTAAGCGCATCAACTGCAT/CTGTCTGACGCTGCAACTGCTA | |
| *vp0219* | AACAAACGACCATTGCTAGG/TGGATTGCCTTCGGTTACGG | |
| *16S* | GACACGGTCCAGACTCCTAC/GGTGCTTCTTCTGTCGCTAAC | |
| **LacZ fusion** | | |
| *cpsA* | GCGCGTCGACCTTCCCTGTAAATAAGTCATCC  /GCGCGAATTCAAGCGAACTCCATCTCATAAG | |
| *scvE* | GCGCGTCGACAAGAGTCTCGTGAACGGATG  /GCGCGAATTCATGGCATCACGACCTGTCTC | |
| *pilA* | | GCGCGTCGACGATACTATCGCTGAATACGCAG  /GCGCGAATTCCAATCAGAGTGAAACCCTGTTG |
| *mshA1* | | GCGCGTCGACTGTTCAGCGTTATCACCAAGC  /GCGCGAATTCATACCAGAAGCACCATCAATCG |
| *scrA* | | GCGCGTCGACCATCAAGCCATTTTATGAAAC  /GCGCGAATTCGTCGGCTGCGATTAGTCTG |
| *scrG* | | GCGCGTCGACTAGCACGCTTGTGTTGGAC  /GCGCGAATTCCAGGGAAATGAAGTAATCATGC |
| *vp0218* | | GCGCGTCGACATGACGCACGCAGTGAATG  /GCGCGAATTCGTTGAAGTTGAATCGCTGTCTG |
| *vp0219* | | GCGCGTCGACCGATGTTTGGTCTGTTTAGAAC  /GCGCGAATTCGGTTACGGAAGAAGTAGAAGAG |
| **EMSA** | | |
| *cpsA* | CTTCCCTGTAAATAAGTCATCC/AAGCGAACTCCATCTCATAAG | |
| *scvE* | AAGAGTCTCGTGAACGGATG/ATGGCATCACGACCTGTCTC | |
| *pilA* | GATACTATCGCTGAATACGCAG/CAATCAGAGTGAAACCCTGTTG | |
| *mshA1* | TGTTCAGCGTTATCACCAAGC/ATACCAGAAGCACCATCAATCG | |
| *scrA* | CATCAAGCCATTTTATGAAAC/GTCGGCTGCGATTAGTCTG | |
| *scrG* | TAGCACGCTTGTGTTGGAC/CAGGGAAATGAAGTAATCATGC | |
| *vp0218* | AACGACACTCTGAGCATCATTC/CGCTGCAACTGCTACTACAG | |
| *vp0219* | CCAGCCATAACTAACACTAACC/AAATGAGCACAAGCCTAAGC | |
| *vp1687* | GCATTATTGACGCCAGTATCG/GGCAACGGTGAGCAAAATC | |
